# Supplementary material for: Lipopolysaccharide-Induced Strain-Specific Differences in Neuroinflammation and MHC-I Pathway Regulation in the Brains of Bl6 and 129Sv Mice
Source: Cells. 2022 Mar 18;11(6):1032. doi: 10.3390/cells11061032 (PMC8946912; doi:10.3390/cells11061032)
Supplement: Supplementary file 1 [file cells-11-01032-s001.zip › cells-1613081-supplementary.pdf]

Supplementary

Maria Piirsalu, Keerthana Chithanathan, Mohan Jayaram, Tanel Visnapuu, Kersti Lilleväli, Mihkel Zilmer and Eero Vasar

Lipopolysaccharide-induced strain-specific differences in neuroinflammatory status and MHC-I pathway regulation in the brains of Bl6 and 129Sv mice

Table S1. Pearson's correlation analysis between 24 h body weight change (ΔBW), body temperature change (ΔBT) and 24 h locomotor activity parameters.

	Pearson r (*p value*)							
	**Bl6 saline**		**Bl6 LPS**		**129Sv saline**		**129Sv LPS**	
	ΔBW	ΔBT	ΔBW	ΔBT	ΔBW	ΔBT	ΔBW	ΔBT
Total distance	0,35 (*0,49*)	0,12 (*0,82*)	-0,19 (*0,75*)	0,40 (*0,51*)	-0,06 (*0,88*)	0,38 (*0,35*)	-0,64 (*0,12*)	0,05 (*0,92*)
Total distance in center	0,47 (*0,34*)	0,29 (*0,58*)	0,19 (*0,76*)	-0,47 (*0,43*)	-0,19 (*0,65*)	-0,13 (*0,76*)	-0,54 (*0,21*)	0,44 (*0,28*)
Time spent moving, total arena	0,22 (*0,68*)	0,21 (*0,69*)	-0,69 (*0,20*)	-0,27 (*0,65*)	-0,42 (*0,30*)	-0,03 (*0,94*)	-0,63 (*0,13*)	0,13 (*0,77*)
Time spent moving, periphery	0,13 (*0,80*)	0,09 (*0,87*)	-0,79 (*0,11*)	-0,15 (*0,81*)	-0,38 (*0,36*)	0,003 (*0,99*)	-0,61 (*0,15*)	0,10 (*0,82*)
Time spent moving, center	0,52 (*0,29*)	0,56 (*0,24*)	-0,37 (*0,54*)	-0,47 (*0,43*)	-0,44 (*0,27*)	-0,30 (*0,46*)	-0,59 (*0,16*)	0,31 (*0,46*)

Table S2. Pearson's correlation coefficient matrices of MHC-I gene expressions and body weight (ΔBW) and body temperature (ΔBT) change in olfactory bulb (OB), hippocampus (HPC), hypothalamus (HT), midbrain (MB), frontal cortex (FC) and cerebellum (CBL) of Bl6 and 129Sv mice. Statistically significant results are indicated in bold ($p \leq 0.05$).

**Bl6 OB**							
	ΔBW	ΔBT	B2m	TapBP	Tap1	Tap2	Lmp2
ΔBW	1	0,54	**0,77**	**0,74**	**0,66**	0,57	0,58
ΔBT	0,54	1	0,60	0,49	0,46	0,55	0,55
B2m	**0,77**	0,60	1	**0,93**	**0,83**	**0,81**	**0,90**
TapBP	**0,74**	0,49	**0,93**	1	**0,91**	**0,94**	**0,91**
Tap1	**0,66**	0,46	**0,83**	**0,91**	1	**0,97**	**0,92**
Tap2	0,57	0,55	**0,81**	**0,94**	**0,97**	1	**0,93**
Lmp2	0,58	0,55	**0,90**	**0,91**	**0,92**	**0,93**	1
**129Sv OB**							
	ΔBW	ΔBT	B2m	TapBP	Tap1	Tap2	Lmp2
ΔBW	1	0,35	-0,14	0,39	-0,19	-0,33	0,06
ΔBT	0,35	1	-0,56	0,04	-0,55	-0,62	0,11
B2m	-0,14	-0,56	1	0,64	0,20	0,53	-0,29
TapBP	0,39	0,04	0,64	1	-0,04	0,24	0,51
Tap1	-0,19	-0,55	0,20	-0,04	1	**0,87**	0,28
Tap2	-0,33	-0,62	0,53	0,24	**0,87**	1	0,11
Lmp2	0,06	0,11	-0,29	0,51	0,28	0,11	1
**Bl6 HPC**							
	ΔBW	ΔBT	B2m	TapBP	Tap1	Tap2	Lmp2
ΔBW	1	0,54	0,08	0,41	0,41	0,51	0,48

ΔBT	0,54	1	0,04	0,35	0,11	0,27	-0,05
B2m	0,08	0,04	1	**0,74**	**0,86**	0,49	0,25
TapBP	0,41	0,35	**0,74**	1	**0,87**	**0,75**	0,15
Tap1	0,41	0,11	**0,86**	**0,87**	1	**0,79**	0,33
Tap2	0,51	0,27	0,49	**0,75**	**0,79**	1	0,06
Lmp2	0,48	-0,05	0,25	0,15	0,33	0,06	1
**129Sv HPC**							
	ΔBW	ΔBT	B2m	TapBP	Tap1	Tap2	Lmp2
ΔBW	1	0,35	-0,10	0,02	0,19	0,18	-0,41
ΔBT	0,35		0,15	-0,49	-0,21	-0,05	-0,47
B2m	-0,10	0,15	1	0,61	0,51	**0,86**	0,41
TapBP	0,02	-0,49	0,61	1	0,58	0,56	0,63
Tap1	0,19	-0,21	0,51	0,58	1	0,57	0,16
Tap2	0,18	-0,05	**0,86**	0,56	0,57	1	0,16
Lmp2	-0,41	-0,47	0,41	0,63	0,16	0,16	1
**Bl6 HT**							
	ΔBW	ΔBT	B2m	TapBP	Tap1	Tap2	Lmp2
ΔBW	1	0,54	0,15	-0,03	0,49	0,41	0,42
ΔBT	0,54	1	0,00	-0,31	0,11	0,09	0,25
B2m	0,15	0,00	1	**0,83**	**0,78**	**0,83**	**0,81**
TapBP	-0,03	-0,31	**0,83**	1	0,64	0,63	0,60
Tap1	0,49	0,11	**0,78**	0,64	1	**0,98**	**0,94**
Tap2	0,41	0,09	**0,83**	0,63	**0,98**	1	**0,94**
Lmp2	0,42	0,25	**0,81**	0,60	**0,94**	**0,94**	1
**129Sv HT**							
	ΔBW	ΔBT	B2m	TapBP	Tap1	Tap2	Lmp2

ΔBW	1	0,35	0,14	-0,01	-0,10	-0,14	-0,10
ΔBT	0,35	1	0,35	0,42	0,17	0,14	-0,05
B2m	0,14	0,35	1	**0,73**	0,61	0,43	0,35
TapBP	-0,01	0,42	**0,73**	1	**0,90**	**0,88**	**0,72**
Tap1	-0,10	0,17	0,61	**0,90**	1	**0,94**	**0,91**
Tap2	-0,14	0,14	0,43	**0,88**	**0,94**	1	**0,91**
Lmp2	-0,10	-0,05	0,35	**0,72**	**0,91**	**0,91**	1
**Bl6 MB**							
	ΔBW	ΔBT	B2m	TapBP	Tap1	Tap2	Lmp2
ΔBW	1	0,54	0,16	0,11	0,62	0,46	**0,74**
ΔBT	0,54	1	0,25	-0,37	0,18	0,21	**0,63**
B2m	0,16	0,25	1	0,38	**0,65**	**0,63**	0,45
TapBP	0,11	-0,37	0,38	1	**0,68**	0,61	0,23
Tap1	0,62	0,18	**0,65**	**0,68**	1	**0,75**	**0,78**
Tap2	0,46	0,21	**0,63**	0,61	**0,75**	1	**0,73**
Lmp2	**0,74**	**0,63**	0,45	0,23	**0,78**	**0,73**	1
**129Sv MB**							
	ΔBW	ΔBT	B2m	TapBP	Tap1	Tap2	Lmp2
ΔBW	1	0,35	0,28	0,27	0,11	0,39	-0,004
ΔBT	0,35	1	-0,06	0,41	-0,27	0,05	-0,22
B2m	0,28	-0,06	1	**0,82**	**0,75**	0,40	**0,80**
TapBP	0,27	0,41	**0,82**	1	0,43	0,33	0,54
Tap1	0,11	-0,27	**0,75**	0,43	1	0,27	**0,96**
Tap2	0,39	0,05	0,40	0,33	0,27	1	0,30
Lmp2	-0,004	-0,22	**0,80**	0,54	**0,96**	0,30	1

**Bl6 FC**							
	ΔBW	ΔBT	B2m	TapBP	Tap1	Tap2	Lmp2
ΔBW	1	0,54	0,00	0,11	0,41	0,31	0,55
ΔBT	0,54	1	0,13	0,21	0,18	0,19	0,44
B2m	0,00	0,13	1	**0,90**	0,06	0,42	0,45
TapBP	0,11	0,21	**0,90**	1	0,48	0,74	**0,77**
Tap1	0,41	0,18	0,06	0,48	1	**0,89**	**0,93**
Tap2	0,31	0,19	0,42	**0,74**	**0,89**	1	**0,91**
Lmp2	0,55	0,44	0,45	**0,77**	**0,93**	**0,91**	1

**129Sv FC**							
	ΔBW	ΔBT	B2m	TapBP	Tap1	Tap2	Lmp2
ΔBW	1	0,35	0,56	**0,73**	**0,71**	0,53	**0,63**
ΔBT	0,35	1	-0,25	-0,18	-0,07	-0,25	-0,13
B2m	0,56	-0,25	1	**0,85**	0,62	0,61	**0,92**
TapBP	**0,73**	-0,18	**0,85**	1	**0,81**	**0,86**	**0,91**
Tap1	**0,71**	-0,07	0,62	**0,81**	1	**0,83**	**0,69**
Tap2	0,53	-0,25	0,61	**0,86**	**0,83**	1	**0,76**
Lmp2	**0,63**	-0,13	**0,92**	**0,91**	**0,69**	**0,76**	1

**Bl6 CBL**						
	ΔBW	ΔBT	B2m	TapBP	Tap1	Tap2
ΔBW	1	0,54	0,44	0,16	**0,65**	0,23
ΔBT	0,54	1	0,54	0,16	0,31	-0,09
B2m	0,44	0,54	1	0,23	**0,81**	0,31
TapBP	0,16	0,16	0,23	1	0,33	-0,16
Tap1	**0,65**	0,31	**0,81**	0,33	1	0,31
Tap2	0,23	-0,09	0,31	-0,16	0,31	1

129Sv CBL						
	ΔBW	ΔBT	B2m	TapBP	Tap1	Tap2
ΔBW	1	0,35	0,06	-0,13	0,07	0,26
ΔBT	0,35	1	-0,09	-0,29	0,35	0,09
B2m	0,06	-0,09	1	-0,02	0,47	0,23
TapBP	-0,13	-0,29	-0,02	1	0,27	-0,25
Tap1	0,07	0,35	0,47	0,27	1	-0,30
Tap2	0,26	0,09	0,23	-0,25	-0,30	1
